# Supplementary material for: Content-rich biological network constructed by mining PubMed abstracts
Source: BMC Bioinformatics. 2004 Oct 8;5:147. doi: 10.1186/1471-2105-5-147 (PMC528731; doi:10.1186/1471-2105-5-147)
Supplement: Additional File 2 — The original results of the above study (non-essential files are deleted to keep the file size under the limit set by BMC bioinformatics). [file 1471-2105-5-147-S2.bz2 › chilibotAdditionalFile2/dip10/173ID9388212E588/html/IL6ST_PTPN11.html]

 


 **IL6ST** and **PTPN11** 
  
Found 54 abstracts in PubMed, retrieved 10.  
 

 What does Google say? 
 PDF only 
| .edu only 

---

**Interactive relationship** (e.g. stimulation, inhibition, etc)

**Neutral relationship**- We show that receptor and membrane targeted SHP2  [ **PTPN11** ]  counteracts IL 6 signaling independent of SOCS3 binding to gp130  [ **IL6ST** ] .  Ref: 12403768 J Biol Chem, 2003
- Taken together, these findings indicate that Gab1 SHP2  [ **PTPN11** ]  interaction plays a crucial role in gp130  [ **IL6ST** ]  dependent longitudinal elongation of cardiomyoctes through activation of ERK5.  Ref: 12855672 Circ Res< MedlineTA>Circ Res, 2003
- Intriguingly, recent in vivo studies indicated that the SHP 2  [ **PTPN11** ]  binding site and YXXQ mediated pathways through gp130  [ **IL6ST** ]  are not mutually exclusive but affect each other a mutation at the SHP 2  [ **PTPN11** ]  binding site prolongs STAT3 activation, and a loss of STAT activation by gp130  [ **IL6ST** ]  truncation leads to sustained SHP 2  [ **PTPN11** ]  ERK MAPK phosphorylation.  Ref: 12687404 Rev Physiol Biochem Pharmacol< MedlineTA>Rev Physiol Biochem Pharmacol, 2003
- These data suggest, that there are two, largely distinct modes of negative regulation of gp130  [ **IL6ST** ]  activity, despite the fact that both SOCS3 and SHP2  [ **PTPN11** ]  are recruited to the same site within gp130  [ **IL6ST** ] .  Ref: 12403768 J Biol Chem, 2003
- Activation of gp130  [ **IL6ST** ]  Transduces Hypertrophic Signal Through Interaction of Scaffolding Docking Protein Gab1 With Tyrosine Phosphatase SHP2  [ **PTPN11** ]  in Cardiomyocytes.  Ref: 12855672 Circ Res< MedlineTA>Circ Res, 2003
- These data suggest that desensitization of vascular cells to OnM is mediated in trans and involves Tyr residue 759 in gp130  [ **IL6ST** ]  but is not mediated by either SOCS 3 or SHP 2  [ **PTPN11** ] , the only two proteins currently known to bind to gp130  [ **IL6ST** ]  at this site.  Ref: 12724316 J Biol Chem< MedlineTA>J Biol Chem, 2003
- We conclude that normal Shp 2  [ **PTPN11** ]  function is critical for the initial step of ES cell differentiation to mesoderm and to hemangioblasts and acts within the LIF gp130  [ **IL6ST** ]  Stat3 pathway to maintain a proper balance of ES cell differentiation, pluripotency, and apoptosis.  Ref: 12791646 Blood< MedlineTA>Blood, 2003
- SHP2  [ **PTPN11** ]  and SOCS3 contribute to Tyr 759 dependent attenuation of interleukin 6 signaling through gp130  [ **IL6ST** ] .  Ref: 12403768 J Biol Chem, 2003
- Tyrosine 759 of gp130  [ **IL6ST** ]  is required for the negative regulation of LIF mediated signaling by both the protein tyrosine phosphatase SHP 2  [ **PTPN11** ]  and the suppressor of cytokine signaling 3 SOCS 3.  Ref: 12425940 Brain Res Mol Brain Res, 2002
- This indicates that STAT signaling is necessary for LIF and CNTF stimulated VIP and ChAT expression and Y759 of gp130  [ **IL6ST** ]  mediates the activities of SHP 2  [ **PTPN11** ]  and SOCS 3, which act to negatively regulate STAT activity.  Ref: 12425940 Brain Res Mol Brain Res, 2002

**Non-interactive relationship** (e.g. studied together, co-existance, homology, etc.)

- Cells transfected with a chimaeric receptor containing the SHP2  [ **PTPN11** ]  selective gp130  [ **IL6ST** ]  intracellular domain showed an enhanced response to cytokine stimulation, which was similar to that shown by a chimaeric gp130  [ **IL6ST** ]  receptor mutant carrying a Y757F point mutation that failed to recruit either SOCS3 or SHP2  [ **PTPN11** ] .  Ref: 12593670 Biochem J, 2003
- Furthermore, activation of p38 mitogen activated protein kinase is shown to be essential for the inhibitory effect of TNF alpha on IL 6 signaling and TNF alpha dependent recruitment of SHP2  [ **PTPN11** ]  to gp130  [ **IL6ST** ] .  Ref: 12817006 J Immunol, 2003
- However, the extent to which hematopoiesis is dependent upon activation of a particular gp130  [ **IL6ST** ]  signaling pathway, namely STAT1 3 or SHP2  [ **PTPN11** ]  MAPK, is unknown.  Ref: 12423677 Exp Hematol, 2002
- ... the Y759 SHP 2  [ **PTPN11** ]  mediated cascade by gp130  [ **IL6ST** ]  stimulation has growth enhancing effects.  Ref: 12687404 Rev Physiol Biochem Pharmacol< MedlineTA>Rev Physiol Biochem Pharmacol, 2002
- On the other hand, SOCS3 inhibits signaling in cells expressing a truncated SHP2  [ **PTPN11** ]  protein, which is not recruited to gp130  [ **IL6ST** ] .  Ref: 12403768 J Biol Chem, 2003
- In the present study, we show the design of a mutant gp130  [ **IL6ST** ]  receptor that can recruit SHP2  [ **PTPN11** ] , but not SOCS3.  Ref: 12593670 Biochem J, 2003
- In this study we demonstrate that stimulation of macrophages and fibroblast cell lines with TNF alpha causes the recruitment of SHP2  [ **PTPN11** ]  to the gp130  [ **IL6ST** ]  signal transducing subunit and leads to tyrosine phosphorylation of SHP2  [ **PTPN11** ]  and gp130  [ **IL6ST** ] .  Ref: 12817006 J Immunol, 2003
- Negative regulation of gp130  [ **IL6ST** ]  signalling mediated through tyrosine 757 is not dependent on the recruitment of SHP2  [ **PTPN11** ] .  Ref: 12593670 Biochem J, 2003
- The subsequent recruitment of the common signal transducer gp130  [ **IL6ST** ]  and activation of the JAK STAT and SHP 2  [ **PTPN11** ]  Ras mitogen activated protein kinase MAPK pathways are responsible for the majority of cellular responses elicited by IL 6 cytokines.  Ref: 12764151 J Biol Chem< MedlineTA>J Biol Chem, 2003
- In this context the cytoplasmic SHP2  [ **PTPN11** ]  SOCS3 recruitment site of gp130  [ **IL6ST** ]  tyrosine 759 is shown to be important for the inhibitory effects of TNF alpha, since mutation of this residue completely restores IL 6 stimulated activation of STAT3 and, consequently, of a STAT3 dependent promoter.  Ref: 12817006 J Immunol, 2003
- Since recruitment of the tyrosine phosphatase Src homology protein tyrosine phosphatase 2 SHP2  [ **PTPN11** ]  to the signal transducing receptor subunit gp130  [ **IL6ST** ]  attenuates IL 6 mediated STAT activation, we were interested in whether TNF alpha also induces the association of SHP2  [ **PTPN11** ]  to the gp130  [ **IL6ST** ]  receptor subunit.  Ref: 12817006 J Immunol, 2003
- In this respect murine fibroblasts lacking exon 3 of SHP2  [ **PTPN11** ]  are not sensitive to TNF alpha, indicating that functional SHP2  [ **PTPN11** ]  and its recruitment to gp130  [ **IL6ST** ]  are key events in inhibition of IL 6 dependent STAT activation by TNF alpha.  Ref: 12817006 J Immunol, 2003
- These results demonstrate that the recruitment of SHP2  [ **PTPN11** ]  alone is not sufficient for Y757 dependent negative regulation of gp130  [ **IL6ST** ]  signalling.  Ref: 12593670 Biochem J, 2003
